# A comparison of formal and informal help in the context of mental health recovery

**DOI:** 10.1177/00207640211004988

**Published:** 2021-03-18

**Authors:** François Lauzier-Jobin, Janie Houle

**Affiliations:** Department of Psychology, Université du Québec à Montréal, Canada

**Keywords:** Personal recovery, therapeutic relationship, social support, helping relationship, agency, deductive thematic analysis, mixed method, critical realism

## Abstract

**Background:**

People in recovery from anxiety, depressive or bipolar disorders can receive both formal (from practitioners) and informal help (from family and friends). These two types of helping relationships have often been studied separately as either therapeutic relationships or social support. Yet, the mechanisms of these two forms of help have not been empirically compared in the context of mental health recovery.

**Aims:**

The purpose of this study is to compare the mechanisms of informal help and formal help in recovery by combining the perspectives of individuals in recovery, their informal helper and their practitioner.

**Method:**

Individual interviews were conducted with 15 triads (*N* = 45 participants) comprising a person in recovery, their most significant informal helper and their most significant practitioner to compare the two forms of help through a mixed method approach. Based on the paradigm of critical realism, the research puts the emphasis on the triangulation of data sources and types.

**Results:**

The informal and formal helping relationships serve multiple functions some can be found in both, often in different ways (communication, presence and availability). Informal helpers tend to serve a broader array of functions than practitioners do. Regarding differences, formal help is characterised by scheduling, time limitations and professional competencies. Informal help is characterised by emotional closeness, companionship and reciprocity. Also, people in recovery are active when it comes to determining the role that their helpers play (agency).

**Conclusions:**

Social support from family members and friends, as well as help from professionals can contribute to recovery in different ways. Attesting to the agency of people in recovery, the two forms of help are not only perceived as complementary, they are deliberately kept so.

## Introduction

In their lifetime, 12% of all people will suffer from a depressive disorder, 11% to 12% from an anxiety disorder and 2.2% from a bipolar disorder (Kairouz et al., 2008; Patten et al., 2006; Schaffer et al., 2006). These mental health problems carry consequences not only for the individuals in question (e.g. Pearson et al., 2013), but also for their families and friends (e.g. Shah et al., 2010) and for society as a whole (Lim et al., 2008).

While trajectories vary across individuals, recovery is not only possible, it is the most likely outcome of their journey (Leonhardt et al., 2017; Slade & Longden, 2015). To recover, they may receive informal help from family and friends, but also formal help from practitioners (Bird et al., 2014; Leamy et al., 2011; Slade, 2012; Tew et al., 2012; Thomas et al., 2018; van Weeghel et al., 2019). However, the majority of people with mental health problems do not consult health professionals (Brown et al., 2014; Clement et al., 2015; Rickwood & Thomas, 2012; Thornicroft, 2007; Wang et al., 2007), preferring the informal help of their social network (Barker & Pistrang, 2002; Brown et al., 2014; Egan, 2013; Winefield, 1987).

As it happens, mental health studies have often focused on formal interventions and neglected informal help, such as social support (Park et al., 2014; Rickwood & Thomas, 2012; Roehrle & Strouse, 2008). Some authors have gone so far as to affirm that ‘no real theory exists of natural helping’ (Stahl & Hill, 2008, p. 291). Others, instead, suggest that social support constitutes a theoretical framework that allows investigating not only informal help, but formal help as well (Huxley et al., 2009; Klauer, 2005; Rickwood & Thomas, 2012). Though theoretical definitions of social support have often excluded professionals (e.g. Gottlieb & Bergen, 2010, p. 512), professionals are an integral part of the social network of people in recovery, like family members and friends (e.g. Pernice-Duca, 2008; Topor et al., 2006). Consequently, the two types of help need to be considered simultaneously (Klauer, 2005; Pescosolido, 2011).

Formal help and informal help (social support) have habitually been treated as distinct variables that influence one another in different ways. First, social support influences both the process of formal help seeking and the use of formal help (Albert et al., 1998; Klauer, 2005). Though results have been mixed, on the whole, strong social support has been shown to reduce recourse to professional help (Chang et al., 2014; Klauer, 2005). Second, for people receiving services, two studies (Calsyn & Winter, 2002; Tsai et al., 2012) have shown the quality support from family and friends to be positively associated with the perceived quality of formal help, though in an another study the association was not significant (Mallinckrodt, 1996). Third, there seems to be a relationship between informal help and psychotherapy. On the one hand, for people in psychotherapy, social support has been shown to have a small-sized positive effect on the psychotherapy outcomes (Roehrle & Strouse, 2008). On the other, in their meta-analysis, Park et al. (2014) reported that psychotherapy had a small- to medium-sized positive effect on the social support that clients received.

Formal and informal help are key factors that promote recovery. These two types of help can be compared from a theoretical perspective on different dimensions, such as their effects, context and processes (Barker & Pistrang, 2002; Klauer, 2005; Winefield, 1987). Different studies found comparable effect size on recovery for formal (Hicks et al., 2012; Kvrgic et al., 2013) and informal helping relationships (Corrigan & Phelan, 2004; Hendryx et al., 2009). Though their effects might be similar, the two types of help differ in terms of contextual variables: such as the helper qualifications (education, training and expertise) or the similarity between the helper and the helpee (Barker & Pistrang, 2002; Klauer, 2005; Winefield, 1987). Finally, some processes of the helping relationship could be similar while others could be specific to each form of help. For example, active listening is thought to be a process common to both, whereas distraction (companionship) exists only in the context of informal help (Winefield, 1987). Research is needed to shed light on the similarities and differences between help provided by family and friends and help provided by practitioners. These two types of help have usually been considered separately and have never been compared empirically in the context of personal recovery.

### The present study

The aim of this study was to compare the core mechanisms of informal help from family and friends and formal help from practitioners in the recovery of people with a depressive, anxiety or bipolar disorder. More specifically, through individual interviews with people in recovery, their family members and friends and their practitioners, we sought to identify the similarities and differences between the two types of helping relationships. We defined mechanisms as an explanation of how relational processes promote recovery in a given context, under given circumstances. Informal helpers were defined as helpers from a person’s informal support network, such as a spouse, a family member, a friend, a co-worker or a neighbour. Practitioners were defined as paid helpers who worked in an institutional or community setting or in private practice. To our knowledge, this was the first study to conduct an empirical comparison of these two types of help in the context of personal recovery.

## Method

A mixed embedded design was used (Creswell & Clark, 2007) whereby a smaller quantitative component (second part of the interview) was meant to support the larger qualitative component. This study is based on the paradigm of critical realism (Emmel et al., 2018; Maxwell, 2012). It placed the emphasis on the triangulation of data sources and types (Clark, 2008; Maxwell, 2012). Developed by Bhaskar (e.g. 2016), critical realism is a philosophy of science that is gaining traction in the social sciences. It is characterised by a reliance on theoretical supports, an explanatory focus (through the notion of mechanisms) and grounding in the real world.

### Procedure

Flyers were distributed by mental health organisations in the Greater Montreal (Quebec) to recruit people in recovery. During a telephone call to verify whether they met the study’s inclusion criteria, people in recovery had to indicate the family member or friend and the practitioner who contributed most to their recovery (see Table 1). We contacted these persons to present the project and verify whether they met the study’s inclusion criteria. Interviews were set when and where convenient for participants. The sampling approach was purposeful and sequential (Patton, 2014). This research project was approved by the research ethics board of the Université du Québec à Montréal. Participants were offered $30 as compensation.

**Table 1.** Participant inclusion criteria.

For people in recovery	For helpers (informal and formal)
(a) 18 years old or over	(a) 18 years old or over
(b) Speak French	(b) Speak French
(c) Able to answer interview questions	(c) Able and willing to answer interview questions
(d) Diagnosed with depression or anxiety at least 12 months prior to interview (self-reported)	(d) Identified by person in recovery as having significantly contributed to their recovery
(e) Consider self in recovery (self-reported)	
(f) Symptoms severity must not be high, as measured on the Patient Health Questionnaire (PHQ-9) and the Generalised Anxiety Disorder Assessment (GAD-7)	(e) Practitioners had to have workplace authorisation to participate in study, if applicable.
(g) Able and willing to designate two significant helpers (one informal and one practitioner) to be interviewed regarding their helper role.	

### Data collection

Individual interviews (*N* = 45) were conducted with people in recovery (*n* = 15), their informal helpers (*n* = 15) and their practitioners (*n* = 15). The first part of the interview consisted of open-ended questions concerning recovery, context of relationship and social support. The second part was more guided and composed of structured questions on 12 functions potentially served by helpers: *to help make decisions*, *to provide emotional support*, *to share attitudes*, *to have trust*, *to provide concrete help*, *to help them back*, *to feel a strong emotional bond*, *to be available*, *to make you feel competent*, *to have power*, *to give advice* and *to instil a sense of belonging*. This list of functions was informed by Cutrona (1989), the Social Provisions Scale (Caron, 1996; Cutrona & Russell, 1987) and our literature review. Respondents had to specify, for their informal helper and for their practitioner separately, whether they served a given function (1) or not (0). See the Supplemental File for the list of questions.

### Data analysis

Interviews were conducted by the first author and analysed with NVivo10. For the first part of the interviews, data were subjected to a hybrid deductive and inductive thematic analysis (Braun & Clarke, 2006). The approach was adapted from Braun and Clarke (2006), Miles et al. (2014), Paillé and Mucchielli (2012), Saldaña (2009) and Zhang and Wildemuth (2009). In the first stage, the interviews were listened to and revised in their entirety by the first author to facilitate phenomenological appropriation. In the second stage, the initial codes were developed based on the conceptual framework. Codes were revisited and a coding manual was created. A first cycle of structural coding of the data was completed. In the fourth stage, a second coding cycle was carried out on the main categories using the margin-tracking thematic analysis method (Paillé and Mucchielli, 2012). In the fifth step, the themes were grouped together, then synthesised and citations were selected for each. In the final stage, the writing, the most relevant themes and the most revealing quotations were retained and integrated into the final writing. Mann-Whitney U tests were run on the IBM SPSS Statistics 26 (Field, 2013) to compare the functions served by informal helpers and those served by practitioners. Average were used to account for missing responses. Given our small sample size, the exact significance method was used and only differences significant at *p* < .10 are reported in the text (see Supplemental File for further details).

## Results

The majority of the participants were women in all three groups: people in recovery (10 women, 5 men), informal helpers (9 women, 6 men) and practitioners (11 women, 4 men). The people in recovery had a mean age of 54.4 years (*SD* = 12.4; min = 30; max = 70) and reported suffering from a bipolar disorder (8), depressive disorder (6) and/or an anxiety disorder (5). Informal helpers were friends (6), spouses (5) or family members (two sisters, one father and one daughter). Six practitioners worked in private practice, six in the public health and social services system and three in community services.

First, the quantitative part of the study revealed that the informal and formal helping relationships served a wide array of functions. Of the 12 functions considered, the people in recovery estimated that their informal helper served 9.9 (*SD* = 2.0) on average, whereas their practitioner served 7.9 (*SD* = 1.7). The difference was statistically significant (*U* = 45.0; *p* = .004).

Of the 12 functions, only three were mentioned more often in connection with the practitioners: *to be available*, *to make you feel good and competent* and *to make interpersonal trust possible*. These three functions were always named in the formal helping relationships (100.0%) but also in most of the informal helping relationships (86.7%, 86.7% and 93.3%, respectively). The other functions were more often served by family members and friends. The difference was particularly pronounced for *to ensure a strong emotional bond* (93.3% for informal helpers vs. 35.7% for practitioners); *to afford possibility of reciprocation* (61.5% vs. 14.2%), *to share attitudes* (66.7% vs. 25.0%) and *to instil a sense of belonging* (86.7% vs. 50.0%). These were the only four statistically significant differences (see Supplemental File for details).

The qualitative analysis allowed us to delve deeper into the similarities and differences between help offered by family and friends and help offered by practitioners. Respondents pointed out differences for the most part: *‘For sure. It’s quite different. It’s very different. Very different’ (person in recovery).* The majority of responses focused on positive aspects of the relationship. The negative aspects were usually the opposite of the positive dimension (e.g. lack of understanding).

The Table 2 summarises the similarities and differences regarding the core mechanisms of help from family and friends and help from practitioners. Various mechanisms were similar but actualised differently according to the type of helping relationship.

*The two can offer emotional support their own way, instrumental support their own way, and informational support their own way as well. Family members and friends and practitioners can provide all the different types of support, but they won’t do it the same way. (practitioner)*

**Table 2.** Similarities and differences between formal and informal helping relationships.

Categories	Similarities and differences	Citations
Presence and availability	Similarity: Family members and friends as much as practitioners were there, present and available.	*“Well, let’s just say that as far as similarities go, being there. [. . .] Being there, being available. Their availability is not the same. [A given practitioner], with her, I had to respect the fact that that my hour was my hour. And to call her otherwise in the event of an emergency. Which happened twice big time. [A given informal helper] I can call him, like, anytime. I could have called him in the evening at midnight and he would have been there if I had needed him then.” (person in recovery)*
	Difference:	
	Continuous presence of family members and friends.	
	Ad hoc presence and availability of practitioners.	
Companionship	Difference:	*“In the formal context, we really need to bear in mind that, seeing how it’s professional, how are we going to be there for the person? Our own personal stuff, that stays pretty much personal. I might say certain things that are public enough or conspicuous enough or things that will really make a difference. But we must always be aware of that with a person.” (practitioner)*
	Fun and shared activities at centre of relationship with family and friends.	
	Practitioner had to set boundaries on this personal dimension.	
Reciprocity	Difference: People in recovery saw their relationship with informal helpers as more symmetrical, mutual and reciprocal; this was rarely the case in their relationship with practitioners.	*“[. . .] it’s a relationship that remains asymmetrical as well. I mean, me, my friends, I hope to have a symmetrical relationship with them. I want to be able to hear what they have to say but I also want them to hear what I have to say. I can see that we’re there for each other, whereas in the therapeutic relationship, I’m there for the other person and the other person is not there for me and that. . . that makes a difference. [. . .] In therapy, I don’t really exist all that much. It is what it is. The relationship is asymmetrical.” (practitioner)*
Communication	Similarities:	*“But, in my opinion, with friends, let’s say, you don’t necessarily talk about the same things that you’ll talk about when you’re in therapy. [. . .] in my case, I’m going through a stretch in my life where, well, I’ve been on work leave for the past eight months and all. When my friends see me it’s “how’re doing”, “how’s it going”, and all that. I try to move. . . to cut short on that, and quickly move on to other things. “Yeah, yeah, doing fine, still going to the support group’ and all that, I give them a little something, a clue about my medication and all that. But [. . .] I don’t want it to go beyond that, no.” (informal helper)*
	Communication rested on active listening and an open, non-judgemental attitude.	
	Speaking and discussions made it possible to ‘grow through understanding’.	
	Differences:	
	Communication was more composed with practitioners and more unfiltered with family members and friends.	
	Topics covered varied according to type of relationship: more delimited with practitioners (vs. talking about this and that with informal helpers), but more in depth.	
Emotional involvement	Differences:	*“When you’re close to someone, anxiety, because you’re close, you know, you’re in it, you sympathise. And it’s hard at times to keep sight of what will really be helpful. Because we have our own fears of the situation. Sometimes you want to insist on something. Sometimes. Whereas, by having that empathy, there’s a certain distance as well. I think that can help.” (practitioner)*
	Informal helpers were more invested emotionally, whereas practitioners empathised (instead of sympathising).	
	Practitioners were outsiders without the same emotional attachment. This distance afforded a sense of safety and freedom that facilitated intervention.	

Aside from the mechanisms described above, formal help differed from informal help in terms of a) scheduling and quality of time; b) framework (professional competencies; deliberate and thought-out; power of influence); and c) agency.

### Scheduling and quality of time

One major difference between relationships with informal helpers and those with practitioners regarded scheduling and the quality of time. With family members and friends, help was part of a natural bond, part of the long term and, often, part of daily life. They could meet the need when it arose. With practitioners, time was limited, and this carried two consequences. First, time became special, it was not part of daily life. This special time was reserved for the helpee, who could then pour themself into it entirely.

*Natural helpers are generally a part of the person’s life, a part of their daily life. Me, I’m there once a week. It’s very different [. . .] Natural helpers, well, they drop by for coffee for an hour every two or three days, so it’s just another moment in her daily life [. . .] when she has an appointment, instead, she’ll wake up two hours earlier to get ready for it. To be sharp. So, it’s something that you notice, yeah. (practitioner)*

The second consequence of time being limited was the structure that this imposed. The help provided by practitioners was delimited in time: *“I come in at a fixed moment and then it comes to an end. With natural helpers, there isn’t necessarily an end to it” (practitioner)*. Actions were geared towards clinical goals and conditioned by organisational considerations: *“The difference is that as a quote unquote professional, I’m accountable, I have therapeutic objectives, objectives in terms of their regaining control of their lives” (practitioner)*. Actions were deliberate and organised, which meant that there was a framework, a game plan, a programme and follow-up on what happened last time and what to expect next time.

### Framework

#### Professional competencies

A major difference lay in the competencies of practitioners that influenced how people in recovery were helped. First, the education, training and knowledge of practitioners served as the basis of their helping behaviours, such as active listening and giving advice. Whereas, not having any training, informal helpers relied on their lived experience and their personal judgement: *“I have no training, I have no qualifications. In fact, all I have to go by is good old common sense and love. It’s simple logic, nothing more” (informal helper)*.

Second, the role played by informal helpers had more to do with being present, attentive and sympathetic, whereas practitioners had the training, knowledge and skills to take it further. This knowledge might relate not only to the client’s problem, but also to the health system and resources. Some of the skills mentioned related to understanding and explaining: question, explore situations organise and links ideas and wrap things up. Other skills related to intervention techniques (e.g. highlighting a person’s strengths or confronting the person) and tools.

#### Deliberate and thought-out

Practitioners helped in a way that was more thought out, which implied, in particular, reflecting upon the help provided, surrounding oneself with a team or receiving supervision: *“They’re trained for that [. . .] the help they provide is deliberate. . . mine is downright unconscious”* (informal helper). A practitioner added:

*I do have that lens there that allows me to see things at another level. That allows me to see what the dynamics and issues are here and now. [. . .] There may be a reason at times why a person doesn’t wish to talk, why they don’t want to move forward and that, in a sense, is the idea, I think, that we may have of being able to step back from the situation. . . we can also name things that are hard to talk about [. . .] and then the capacity that I have, me, as a professional, to obtain support from the members of the team. (practitioner)*

For their part, informal helpers might not have a choice whether to help or might be ill equipped to do so, which could have consequences for them as informal helpers (e.g. feeling incompetent, getting angry). Some practitioners emphasised this point and offered help to family members and friends.

#### Power of influence

Thanks to their education and training, their knowledge and their competencies, practitioners had a greater power of influence than family members and friends did: *“She’ll pay more attention to what the practitioner might say than to what I might say. Even though, oftentimes, I might say the exact same thing as the practitioner”* (informal helper). This greater power of influence brought about a certain degree of restraint on the part of practitioners: *“you need to pay more attention to what you say” (practitioner)*. This restraint was not just a question of attitude: It also rested on legal and ethical considerations whereby professionals were recognised as qualified to exercise their practice and accountable.

### Agency

People in recovery intentionally called upon the two types of relationships in different ways. They chose what to ask one source of support or the other. These sources were considered as complementary and were deliberately kept so. One person in recovery opined the following:

*As far as differences go, [my psychologist] is a professional. She maybe has more resources than [my sister] does but that’s okay because if my sister turned into a psychologist, I don’t think that would be a good thing for me. [. . .] I’d see her in a different light. If my sister was a psychologist and if. . . it wouldn’t be the same. I rather have a psychologist and a sister. (person in recovery)*

Similarly, one practitioner underscored that different relationships could encourage different types of disclosure, without one source being better than the other.

*[. . .] family members and friends can offer things that practitioners cannot. I find it interesting that the two roles not be too similar, that they should complement one another instead. Family members and friends, they have a bond that practitioners will maybe never have with the person. So it depends on the relationship between family members and friends and the mentally ill person, and the quality of the relationship. Even in terms of disclosure, it can go either way. At times the helped person might feel more comfortable disclosing stuff to their practitioners than to family members or friends, and vice versa. (practitioner)*

## Discussion

To our knowledge, our study is the first to compare empirically the mechanisms of formal and informal help in the recovery of people with depressive, anxiety and bipolar disorders. Our results show that helping relationships, be they formal or informal, serve numerous functions in mental health recovery. Certain conclusions can be drawn regarding their similarities and differences.

The two types of helping relationship serve multiple functions, although in different ways. For example, they share communication and active listening but actualise these differently. The two types of help are based on active listening, a non-judgemental attitude and discussions, which are conducive to “*growing through understanding*” (person in recovery). However, because of the formal context of the relationship, communication is more subdued and targeted with practitioners and unfiltered and spontaneous with family members and friends. These results are in line with and refine and empirically support the theoretical intuitions of previous theoretical studies on this subject (Klauer, 2005; Winefield, 1987).

Informal helpers serve a broader array of functions than practitioners do. The difference between the two is especially striking concerning emotional bonds, reciprocity and companionship. With family members and friends, fun and shared activities are at the centre of a relationship that is more symmetrical and reciprocal.

Three differences in the mechanisms of formal and informal help are particularly noteworthy. First, as pointed out by Winefield (1987) under the notion of attachment, practitioners keep a certain emotional distance. This detachment instils a sense of safety in the person in recovery and facilitates the therapeutic work (sharing, listening, reflecting and confronting). Professionals maintain this distance by establishing a framework and professional boundaries and by making the distinction between empathy and sympathy.

A second difference regards interpretation. This, too, was noted by Winefield (1987): ‘The defining characteristic of psychotherapy has been claimed to be interpretation’ (p. 634). Under the category of *professional competencies*, we advance that the education, training and knowledge of practitioners serve as the basis for their help behaviours. Informal helpers, instead, rely on their personal experience and judgement. Professionals have the competencies to take their help further, whether in terms of understanding, explaining or intervention techniques and tools.

Third, unlike informal helpers who do not always have a choice whether to assume their helper role, practitioners chose freely to become professional helpers, though they might not always wield control over all the aspects of this role (e.g. they do not always choose their clients or the mode of intervention). The help provided by professionals is more deliberate and thought out. It is therefore easier for them to step back and question the dynamics of the helping relationship and to seek support (e.g. team, supervision), if necessary.

Our results show also that people in recovery are not passive when it comes to determining the role that their helpers play. For example, they choose what to talk about with one or the other source of support. Not only are the two forms of help considered complementary, they are deliberately kept so. People in recovery retain power or agency in their helping relationships, as underlined by Perry and Pescosolido (2015). It may also be that people in recovery purposefully try not to ask too much of their family members and friends for fear of becoming a burden on them or of coming across as negative all the time. Because of the therapeutic framework, people in recovery may feel less need to regulate their disclosure (Milne, 1999).

### Implications

Regarding research, one implication of our study concerns the importance of taking formal and informal help into account simultaneously. In both studies and interventions, it is essential to recognise that recovery does not occur in a vacuum but through a wide array of interpersonal and social interactions (Mezzina et al., 2006; Rose, 2014; Topor et al., 2006; Wyder & Bland, 2014). More specifically, future research should consider help provided by the social network (family, relatives and friends) and by practitioners at the same time. These different sources of support can have a different influence on personal recovery. Research on mental health help seeking pathways, too, must consider the different sources of help. In a literature review, Rickwood and Thomas (2012) found that only one-third of the studies of help seeking took account of informal help.

Regarding intervention, we encourage practitioners to consider professional help within the context of the client’s life as a whole, which means taking account of their informal help and the self-management strategies that they may have tried out in the past. A person’s social support will influence various aspects of professional intervention: help seeking, motivation, intervention perseverance and outcomes, including recovery (Klauer, 2005; Milne, 1999; Roehrle & Strouse, 2008). It is important, then, to systematically measure the social support received by persons when intervention begins (Caron & Guay, 2005; Cohen et al., 2000; Milne, 1999). Aside from the direct evaluation of a person’s social support, two other interesting possibilities are worth considering: the systematic integration of feedback and the evaluation of systemic alliance.

First, client feedback allows professionals to adjust their interventions and ultimately improve outcomes, particularly in cases where only limited progress is being made (Lambert & Shimokawa, 2011; Lambert et al., 2019). This is a procedure already used in different psychotherapy approaches. Soliciting information on a regular basis on the therapeutic relationship and on the client’s relational situation can allow professionals to adjust their interventions. To this end, Lambert et al. (2019) developed the Clinical Support Tool (CST), which allows measuring progress in psychotherapy, flagging not-on-track cases and identifying eight potential problem areas, the first three of which are the therapeutic alliance, motivation and social support.

Second, though social support is the concept most commonly used to describe the help provided by family members and friends, it is not the only one. Systemic alliance is a concept that encompasses not only the therapeutic alliance between client and practitioner, but also the alliance between client and, respectively, spouse, family and social network, as applicable, even if they are not present physically during intervention. Pinsof et al. (2008) developed the Individual Therapy Alliance Scale (ITAS) to measure this broader notion. The CST and the ITAS are interesting instruments for taking account of the social network of persons in psychotherapy. Unfortunately, neither has ever been used in the context of mental health personal recovery.

As informal help precedes, co-occurs with and survives formal help, social support turns out to be an essential extra-therapeutic factor to consider in interventions. The complementarity between the two forms of help becomes a key lever in planning and preparing the end of an intervention. A proper evaluation would allow identifying who in the social network already serves various social support functions, which functions are lacking and which might be developed further or served by another source of support.

### Limitations

This study has limitations that need to be pointed out. First, the diagnoses of anxiety, depressive and bipolar disorder were self-reported by the people in recovery. Second, formal and informal helpers were recruited by referral: These were the individuals who had most contributed to recovery as a result the diversity functions of caregivers may have been overestimated. Caution must be exercised in generalising the results to other health problems and other forms of support. Finally, the quantitative results are of an exploratory nature given the small sample size and the absence of the use of standardised tools.

## Conclusion

Personal recovery is not achieved in a vacuum. The members of the person’s social network can contribute to it in different ways. Social support from family members and friends, as well as help from professionals are complementary forms of help that, when considered simultaneously, can influence and enrich one another. Attesting to the agency of people in recovery, these two forms of help are considered and also kept as complementary. Finally, it is crucial to explore the requisite conditions for formal and informal helpers to fulfil their essential roles as effectively as possible.

## Supplemental Material

sj-pdf-1-isp-10.1177_00207640211004988 – Supplemental material for A comparison of formal and informal help in the context of mental health recovery Click here for additional data file. Supplemental material, sj-pdf-1-isp-10.1177_00207640211004988 for A comparison of formal and informal help in the context of mental health recovery by François Lauzier-Jobin and Janie Houle in International Journal of Social Psychiatry
