# Supplementary material for: A comparison of formal and informal help in the context of mental health recovery
Source: Int J Soc Psychiatry. 2021 Mar 18;68(4):729–37. doi: 10.1177/00207640211004988 (PMC9014766; doi:10.1177/00207640211004988)
Supplement: sj-pdf-1-isp-10.1177_00207640211004988 – Supplemental material for A comparison of formal and informal help in the context of mental health recovery [file sj-pdf-1-isp-10.1177_00207640211004988.pdf]

Supplementary file

The second part of the interview was more directive and allowed for a comparison of the functions performed by the caregiver and the practitioner. Table 3 shows the questions for the person in recovery, the caregiver and the practitioner.

Table 3 : Questions for the second part of the interview

Items	Questions to the person in recovery	Questions for the helper (caregiver or practitioner)
1	Who can you talk to if you have important decisions to make?	Do you think [person] comes to talk to you when they have important decisions to make?
2	Who are the people you can confide in, talk openly about your problems and receive emotional support?	Does [person] confide in you? Does [person] talk to you about their problems?
3	Who are the people who share your attitudes, beliefs, interests and concerns?	Do you share [person]'s beliefs, attitudes, interests and concerns?
4	Who do you trust?	Do you believe [person] trusts you?
5	Who are the people you can count on to provide you with concrete and material help, especially when you are in trouble?	Do you provide concrete and material help to [person]?
6	Who allows you to help them in return?	Does [person] help you in turn?
7	With whom do you feel a strong emotional bond?	Do you feel that you have a strong emotional connection with [person]?
8	Who are the people who are present and available for you?	Do you consider yourself present and available to [person]?
9	Who makes you feel good and competent? Who emphasizes your strengths and abilities?	Do you acknowledge [person]'s strengths and abilities?
10	In what relationship do you have power, for example, over decision-making?	In your relationship with [person], who has the power, for example, in decision-making?
11	From whom do you seek advice and perspectives?	Do you give [person] advice or perspectives?
12	Who are the people who make you feel included or give you a sense of belonging?	Do you feel that you give [person] a sense of inclusion or belonging?

Mann-Whitney U tests were run on the IBM SPSS Statistics 26 (Field, 2013) to compare the functions served by informal helpers and those served by practitioners. Average were used to account for missing responses. Table 4 shows the frequency of the functions served by informal helpers and those served by practitioners as well as the comparison between both from the perspective of the person in recovery.

Tableau 4 : Perceived frequency and comparison of functions by relationship (according to the recovering person)

Items (short)	Relationship with the caregiver	Relationship with the practitioner	Mann-Whitney U	Exact significance (p)[1]
1. Help make decisions	86.7%	80.0%	105.000	0.775
2. Provide emotional support	100.0%	85.7%	90.000	0.533
3. Share attitudes	66.7%	25.0%	52.500	0.067
4. Have trust	93.3%	100.0%	98.000	0.78
5. Provide concrete help	66.7%	57.1%	95.000	0.683
6. Help them back	61.5%	14.3%	48.000	0.038
7. Feel a strong emotional bond	93.3%	35.7%	44.500	0.007
8. Be available	86.7%	100.0%	97.500	0.539
9. Make you feel competent	86.7%	100.0%	91.000	0.561
10. Have power	73.3%	58.3%	76.500	0.516
11. Give advice	80.0%	73.3%	105.000	0.775
12. Instil a sense of belonging	86.7%	50.0%	66.500	0.093
		Total number of functions	45.000	0.004

[1] Following the recommendation of Field (2013), the exact significance method is reported because of the small sample size.
